# Editorial: Health technology assessment in cardiovascular diseases

**DOI:** 10.3389/fcvm.2023.1108503

**Published:** 2023-01-24

**Authors:** Komal Shah, Kamal Sharma, Deepak Saxena

**Affiliations:** ^1^Department of Public Health, Indian Institute of Public Health Gandhinagar, Gandhinagar, Gujarat, India; ^2^Department of Cardiology, SAL Hospital, Ahmedabad, India

**Keywords:** health technology assessment (HTA), cardiovascular diseases, healthcare technologies, innovations, policy maker decision support system, evidence-based medicine

According to World Health Organization (WHO), cardiovascular diseases (CVD) remains the number one killer in developing as well as developed countries, accounting for 17.9 million deaths each year (32% of the total deaths), with an expected increase to 23.6 million by 2030^1^. With the chronic nature of the condition, CVD not only has long-term clinical consequences but also a substantial economic burden on both health systems and patients. The CVD cost globally was approximately US$ 863 billion in 2010 and is estimated to rise by 22% (around US$ 1,044 billion) by 2030 (1). Cost estimate for coronary heart disease (CHD) and stroke can be as high as 5,000 US$ per episode (2). Moreover, an epidemiological shift with the disease affecting the younger population at large has made the situation more alarming. These have paved the way for investing in inventing, designing, and implementing approaches and healthcare technologies (HCTs) for improving diagnosis, treatment, and prognosis of CVD with substantial efforts being directed toward the assessment of these technological support solutions.

Over the last few decades, the advancement in medical technologies has touched almost all spheres of life with health care innovations aiding clinicians and health care professionals (HCP) in decision making. HCTs have become one of the fastest growing global markets with an estimated size of 857.58 US$ billion by 2030^2^. Unfortunately, adopting these new technologies can put a huge financial burden on the healthcare system. Hence, it is imperative to identify and adopt technologies and interventions which can contribute significantly to the prevention and management of the disease and provide economically, socially, and contextually acceptable alternatives through systematic, ethical, unbiased, transparent, and robust methods of innovation. These evidence-supported approaches can guide policy decisions for both developing and developed economies. The goal is to support the interventions for a sustainable health system. Health Technology Assessment (HTA) is a relatively new concept in developing countries. It is defined as a form of policy research that supports an evidence-based decision for the use of any technological innovation aiming to improve health outcomes. It recommends the incorporation of innovation in the policy on the bases of clinical efficacy, economic effectiveness, and social and ethical implications of the HCTs. Areas in which HTA could be applied in the state and national contexts are new equipment procurement, new drug procurement, development of clinical practice guidelines/protocols for common diseases, screening of societal problems for the development of products/processes/prototypes, and prioritizing interventions that represent the greatest value to address the problem within a limited budget.

As per an estimate, the CVD technology market will exceed 40US$ billion by 2030^3^. The HCTs shall form the basis of innovations in all the domains of the solutions ranging from imaging (CT, PET, and MRI), AI-supported prediction and diagnostic tools, genetic and novel biomarkers, non-invasive tools, novel surgical approaches, and drug discovery, to name a few. Along with the rise in the global burden of the disease, there is an expected proportionate increase in the market size and hence the need for rational decision-making with regard to the choice of the solution adopted by the HCPs. HTA is an extension of evidence-based medicine (EBM), critically considering cost-effectiveness analysis with ethical and societal factors into consideration, making it a suitable research method for tailoring contextual policy decisions.

Understanding the need for HTA, various countries have adopted resolutions on linking HTA evaluation with the decision-making process. Countries have established their own administrative bodies for HTA which guide, conduct, and recommend HTA studies based on the felt need of the population and health care budgets of their country and/or the state. The WHO has also directed efforts to broaden the use of HTA across the globe including achieving the sustainable development goal of universal health coverage^4^.

This Research Topic covers health technology assessment topics in the field of cardiovascular diseases. The authors have presented their work in the area of CVD technologies and programs that include innovations, medical technologies, programs, and drugs.

The use of artificial intelligence (AI), machine learning (ML), and deep learning (DL) is an important application of data sciences in medical sciences, especially in HTA. In this edition, the use of ML is evaluated for predicting hypotension while DL is used in contextual setup for diagnosing atrial fibrillation. ML is evaluated across 3 countries for predicting the development of hypertension in another original study. A study by Islam, Talukder et al. applies six common ML-based classifiers viz. decision tree (DT), random forest (RF), gradient boosting machine (GBM), extreme gradient boosting (XGBoost), logistic regression (LR), and linear discriminant analysis (LDA) to predict hypertension and its risk factors in 8,18,603 participants, of which 82,748 (10.11%) had hypertension. The ML models perform well to predict hypertension and its associated factors in South Asians. If these models are employed on an open-source platform, they can be scaled up to benefit millions of patients.

Kumar et al. in the CACHET-CADB study, was a subpart of the REAFEL study to optimize the diagnosis of atrial fibrillation using an AI/ML-based algorithm. In contrast to the existing databases apart from the ECG, CACHET-CADB also provides the continuous recording of patients' contextual data such as activities, body positions, movement accelerations, symptoms, stress level, and sleep quality. This can help to develop and evaluate algorithms for patient-operated wearable ECG, thereby making longitudinal ambulatory monitoring more economically robust and feasible. Similarly, another study conducted by Zhao A. et al., provides a systematic summary of articles providing the accuracy of ML-based algorithms for predicting acute hypotension. The authors report the accuracy of algorithms from 70–96% with a sample size ranging from 12–3,825 participants identified from 35 original studies.

A review from the Himalayan country of Nepal delves into the felt need of the same (Adhikari et al.). The use of a modified Delphi approach in a smart home system to improve the self-management behavior of the patients is explored. The prevalence of DM (4.4–18.8%) and HTN (17.2–70.0%) is reported in most of the studies, covering the other aspects of HTA of DM/HTN. Islam, Nourse et al. provide consensus-based recommendations for functions of a smart home system for self-management of chronic heart failure in the community. Through the Delphi method, a consensus is obtained among 15 experts through two rounds of discussion, and a 34-item smart home system is designed to support people with chronic heart failure for self-management and clinical support.

A cost-effectiveness analysis of pharmacist-managed warfarin therapy in patients with prosthetic valves was evaluated in the Egyptian population which has the potential to bring the down the costs of patient care (Batran et al.). The pharmacist-managed warfarin therapy strategy is proven to provide a significantly better anticoagulation control and to be a cost-saving approach in Egyptian patients.

Electrography-based technologies are explored for the prediction of a variety of cardiovascular conditions. The use of speckled tracking in echocardiography has the potential to predict coronary artery disease and in research from Taipei Medical University, Chaichuum et al. attempts to show a correlation between average longitudinal strain and SR of the segmental myocardium supplied by 170 coronary arteries by calculating vessel myocardium strain (VMS) and strain rate (VMSR) to predict significant coronary artery stenosis in angiographically proven CAD patients. A VMS and VMSR higher than 16.9 ± 4.9 and 1.2 ± 0.3, respectively, predicts mild or no coronary artery stenosis. Zhao T. et al., show that electrocardiographic indicators can be useful for the individualized treatment of pediatric vasovagal syncope (VVS). Various ECG indicators such as HRV, Pd, QTd, Tp-Te interval, Tp-Te/QT, T-wave amplitude and shape, immediate heart rate change, DC, and VLP can be useful tools for VVS due to their easy availability and cost-effective nature. Similarly, Guo et al. show the potential utility of electrocardiographic variables for screening for hypertrophic cardiomyopathy (HCM). From 423 patients' data, 30 ECG features were studied and all except an abnormal Q wave are found to be significantly different between the HCM patients and non-HCM patients. A model using two ECG features - T wave inversion (TWI) and the amplitude of S wave in lead V1 (SV1) was developed for HCM prediction and it is found to have good discrimination power (c-statistics of >0.75). Another study (Tang et al.) shows a correlation between QT interval on ECG with depression and anxiety. The study uses data from 61 patients from China and suggests the potential utility of QT interval for the prediction of depression and anxiety.

Jiang et al. in their research, show how the use of Galectin-3 can be used to predict hospitalization in heart failure with preserved ejection fraction (HFpEF) with a moderate diagnostic value (AUC: 0.763) which is better than that of interventricular thickness or E/A ratio. A novel method of Pulsed field ablation (PFA) is studied and discussed by Bi et al.. This preclinical study finds that biphasic asymmetric pulses can reduce muscle contractions and drop ablation threshold and that this method can be adapted due to its safe and cost-effective outcomes.

Another article (3) discusses the role of a left ventricular assist device as a destination therapy and comments on its cost-effectiveness in the United Kingdom. The authors suggest that it can be an economic alternative considering the limited availability of donor organs for heart transplants. Two other commentaries [(4); Rawal et al.] discuss the role and cost-effectiveness of statin for primary prevention and Rivaroxaban with or without aspirin in coronary artery disease patients.

The advancement in healthcare technologies is headed toward a major revamp with data sciences and healthcare technology assessment (HTA) driving the next potential revolution in not only early diagnosis and treatment but also in prognostication and primordial prevention in times to come and this edition on HTA tries to envisage and bring the same to the forefront.

## Author contributions

All authors listed have made a substantial, direct, and intellectual contribution to the work and approved it for publication.

## Footnotes

^1^ https://www.who.int/en/news-room/fact-sheets/detail/cardiovascular-diseases-(cvds)

^2^ https://www.ibef.org/industry/information-technology-india

^3^ https://www.dicardiology.com/content/cardiovascular-disease-technology-market-will-exceed-40-billion-2030

^4^ https://www.who.int/teams/health-product-policy-and-standards/assistive-and-medical-technology/medical-devices/assessment
